# A national study of socioeconomic status and tuberculosis rates by country of birth, United States, 1996–2005

**DOI:** 10.1186/1471-2458-12-365

**Published:** 2012-05-18

**Authors:** Nicole A Olson, Amy L Davidow, Carla A Winston, Michael P Chen, Julie A Gazmararian, Dolores J Katz

**Affiliations:** 1 California Department of Public Health, STD Control Branch, Richmond, CA, USA; 2 Department of Preventive Medicine, New Jersey Medical School, Newark, NJ, USA; 3 Centers for Disease Control and Prevention, Division of TB Elimination, Atlanta, GA, USA; 4 Emory University, Rollins School of Public Health, Atlanta, GA, USA

## Abstract

**Background:**

Tuberculosis (TB) in developed countries has historically been associated with poverty and low socioeconomic status (SES). In the past quarter century, TB in the United States has changed from primarily a disease of native-born to primarily a disease of foreign-born persons, who accounted for more than 60% of newly-diagnosed TB cases in 2010. The purpose of this study was to assess the association of SES with rates of TB in U.S.-born and foreign-born persons in the United States, overall and for the five most common foreign countries of origin.

**Methods:**

National TB surveillance data for 1996–2005 was linked with ZIP Code-level measures of SES (crowding, unemployment, education, and income) from U.S. Census 2000. ZIP Codes were grouped into quartiles from low SES to high SES and TB rates were calculated for foreign-born and U.S.-born populations in each quartile.

**Results:**

TB rates were highest in the quartiles with low SES for both U.S.-born and foreign-born populations. However, while TB rates increased five-fold or more from the two highest to the two lowest SES quartiles among the U.S.-born, they increased only by a factor of 1.3 among the foreign-born.

**Conclusions:**

Low SES is only weakly associated with TB among foreign-born persons in the United States. The traditional associations of TB with poverty are not sufficient to explain the epidemiology of TB among foreign-born persons in this country and perhaps in other developed countries. TB outreach and research efforts that focus only on low SES will miss an important segment of the foreign-born population.

## Background

As populations have migrated from countries with high tuberculosis (TB) rates to countries with low TB rates [1], the proportion of TB cases in the United States diagnosed among foreign-born persons has steadily risen, from less than 25% in 1986 to more than 60% in 2010 [2,3]. While TB rates declined in both foreign-born and U.S.-born populations over this period, the decline was faster and more consistent among the U.S.-born; as a result, the TB rate ratio for foreign-born compared to U.S.-born persons rose from 3.8 in 1990 to 11.3 in 2010 [3,4].

Most TB cases among foreign-born residents of the United States are due to reactivation of latent tuberculosis infection (LTBI) acquired in their countries of origin, where TB rates may be more than 60 times higher than the TB rate in the United States [5-7]. A study based on tuberculin skin testing in the 1999–2000 National Health and Nutrition Examination Survey estimated that 6.9 million foreign-born persons living in the United States had positive tuberculin skin tests [8]. As these persons constitute the reservoir for the majority of TB cases diagnosed annually in the United States, control of TB in the United States will increasingly depend on identifying and providing preventive treatment to foreign-born persons with LTBI [9,10].

Effective targeted testing and prevention efforts require an understanding of the demographics of targeted populations, including factors such as socioeconomic status (SES).

While TB historically has been associated with poverty [11], three previous studies have suggested that foreign-born persons in the United States may differ from U.S.-born persons in this respect [12-14]. All three studies had limitations; the only national study focused primarily on race/ethnicity and addressed country of origin only incidentally [12], while the other two were limited to single jurisdictions [13,14]. Moreover, these studies used data from 1987–1999, when foreign-born persons represented a minority of TB cases in the United States. Only in 2002 did the proportion of TB cases among foreign-born persons exceed 50% [3]. It is important to know how these changes in the epidemiology of TB in the United States have affected the associations of TB and SES in U.S.-born and foreign-born populations.

Our study used TB data from a more recent time period (1996–2005) to calculate and compare rates of TB in U.S.-born and foreign-born populations by population measures of SES. We hypothesized that while TB remains strongly associated with low SES in the U.S.-born population, this association is more complex among the foreign-born and varies by country of birth. This hypothesis was examined by linking TB cases reported to a national surveillance database with ZIP Code-level measures of SES from the U.S. Census 2000.

## Methods

### Data sources and population

Information on TB cases in the United States was obtained from the Centers for Disease Control and Prevention’s National Tuberculosis Surveillance System (NTSS), which includes records of all verified TB cases reported since 1993. Since this analysis used population data from U.S. Census 2000, TB cases reported in the decade range 1996–2005 were chosen to bracket the census year. All case records selected included country of origin, ZIP Code of residence, and date when the health department verified the TB case. The NTSS defines U.S.-born as someone born in the United States, to a U.S. citizen overseas, or in a U.S. associated jurisdiction (e.g., Puerto Rico, Guam, etc.). All other persons are defined as foreign-born [15]. The NTSS has been determined to be routine public health surveillance, not human participant research requiring oversight by an institutional review board. Data are reported by state and local health departments and CDC does not receive any information that allows direct identification of patients, such as names.

Population denominator data and information on SES were obtained from the U.S. Census 2000, Summary File 3, collected at the ZIP Code Tabulation Area (ZCTA) level. ZCTAs were created by the U.S. Census Bureau to follow census block boundaries and are similar to the ZIP Codes used by the U.S. Postal Service [16,17].

Four ZCTA-level variables that reflect SES and were used in previous studies of TB and SES were chosen: (1) education, defined as the percentage of the population 25 and older with any college attendance, (2) unemployment, defined as the percentage of the total population in the civilian labor force that is unemployed, (3) crowding, based on the U.S. Census definition of the percentage of housing units with more than one person per room [18], and (4) income, based on per capita income in 1999 [12-14]. Total population counts were available in the U.S. Census 2000 by nativity for all ZCTAs, allowing separate rates to be calculated for U.S.-born, foreign-born, and specific countries of origin.

The analysis included TB cases reported from 1996 through 2005 with ZIP Codes that matched U.S. Census ZCTAs. ZCTAs with small populations (<100) were excluded for statistical stability.

### Statistical analyses

ZCTAs were rank ordered by percentage values for each of the four SES variables and grouped into quartiles, with each ZCTA containing at least one resident TB case contributing once. Thus, each SES variable could have different ZCTAs in its quartiles. ZCTAs with higher percentages of college attendance and per capita income, and lower percentages of unemployment and crowding, were considered to have higher SES. These area-level SES quartile groupings were calculated for ZCTAs overall, so foreign-born and U.S.-born persons in the same ZCTA would share the same SES level for each SES variable.

For each quartile grouping of an SES variable, 10-year TB rates were calculated by ZCTA separately for U.S.-born persons, for foreign-born persons overall, and for foreign-born persons from each of the five countries with the highest TB case counts in the United States: Mexico, the Philippines, Vietnam, India, and China [4]. Case counts from the NTSS database and population counts by birth countries from the U.S Census 2000 were used to calculate rates. Spearman’s coefficients assessed pair-wise correlations between the SES variables.

Poisson regression models examined the relationships between TB rates and ZCTA SES-related variables, stratified by country of origin in order to assess the contribution of the SES variables separately for U.S.-born and foreign-born persons. As the purpose of the analysis was to assess the main effect of each of the SES variables on TB rates adjusting for the confounding effects of the other variables, the four variables were retained in all analyses. The models did not include individual level variables such as age and gender because the outcome measure was ZCTA-level TB rates.

The generalized estimating equation approach was used to account for clustering within each ZCTA [19]. To simplify presentation and avoid the need for complex interaction terms, the quartiles used in the bivariate analyses were reduced to two groups in the multivariate analysis, with the two higher quartiles classified as high SES and the two lower quartiles classified as low SES. Because the reduction to two groups might mask the strength of association between the highest and lowest quartiles, a sub-analysis excluded records with values in the middle two quartiles for all four SES variables. All analyses were conducted with SAS version 9.2 (SAS Institute, Inc., Cary, NC).

## Results

Of the 170,590 verified TB cases in the United States from 1996 to 2005, 143,579 (84.2%) had complete ZIP Codes, corresponding ZCTAs in the census dataset, known country of origin (U.S.-born or foreign-born), and ZCTA populations of at least 100 persons. Almost 96% of foreign-born cases matched a ZCTA, compared to 75% of U.S.-born cases.

Approximately half (52.2%) of the cases included in the analysis were in foreign-born persons. U.S.-born persons with TB were more likely than foreign-born persons to be male (64.3% and 59.2%, respectively), and were older (mean age 46.8 and 42.8, respectively).

The ranges of values for each SES quartile are shown in Table 1. Pair-wise correlation coefficients between SES variables were all ≥0.34. Education and income were highly correlated (correlation coefficient = 0.78), and unemployment and income were moderately correlated (0.67).

**Table 1:** Quartile distributions of U.S. Census SES variables in ZIP Codes with TB cases, 1996–2005

	**Cut-points (%) or ($)**
**Percent of population 25 and over that attended any college (Education)**	
1 – Low SES	0 – 39.4
2	39.5 – 51.5
3	51.5 – 65.9
4 – High SES	65.9 – 100.0
**Percent of housing units with one or more persons per room (Crowding)**	
1 – Low SES	7.6 – 65.6
2	3.7 – 7.6
3	1.8 – 3.7
4 – High SES	0 – 1.8
**Average per capita income in 1999 (Income)**	
1 – Low SES	3729 – 16 016
2	16 017 – 20 040
3	20 041 – 26 224
4 – High SES	26 225 – 114 359
**Percent of population in the civilian workforce that was unemployed in 1999 (Unemployment)**	
1 – Low SES	7.5 – 83.9
2	5.0 – 7.5
3	3.5 – 5.0
4 – High SES	0 – 3.4

### Bivariate analyses

Among both foreign-born and U.S.-born persons, higher TB rates were associated with lower SES. The highest average TB rates were in the areas with the lowest SES for all four SES-related variables, with TB rates increasing from the highest to the lowest SES quartile (Table 2). TB rates were higher among the foreign-born overall and within each SES-related measure.

**Table 2:** TB rates by U.S. Census SES variables for foreign-born and U.S.-born persons, 1996–2005

	**TB rate per 100 000**		
	**Foreign-Born**	**U.S.-Born**	**Rate Ratio**
	(95% CI^*^)	**(95% CI)**	**(95% CI)**
	**(n = 75,246)**	**(n = 68,333)**	
**Overall**	25.8 (25.3, 26.4)	3.94 (3.91, 3.98)	6.55 (6.47, 6.63)
**Education**^**†**^			
1 (Low SES)	27.2 (26.2, 28.3)	8.18 (8.06, 8.30)	3.33 (3.26, 3.41)
2	27.9 (26.7, 29.0)	4.31 (4.24, 4.39)	6.46 (6.31, 6.61)
3	26.0 (24.8, 27.2)	2.65 (2.60, 2.70)	9.82 (9.57, 10.1)
4 (High SES )	21.6 (20.5, 22.7)	1.71 (1.68, 1.75)	12.6 (12.2, 13.0)
**Crowding**^**‡**^			
1 (Low SES)	27.5 (26.5, 28.5)	7.66 (7.54, 7.78)	3.59 (3.51, 3.66)
2	25.0 (23.9, 26.2)	4.12 (4.04, 4.19)	6.08 (5.92, 6.25)
3	23.1 (21.9, 24.3)	2.30 (2.26, 2.35)	10.0 (9.72, 10.3)
4 (High SES )	19.4 (18.3, 20.6)	1.31 (1.27, 1.34)	14.9 (14.4, 15.4)
**Income**^**§**^			
1 (Low SES)	28.1 (27.1, 29.1)	8.83 (8.70, 8.96)	3.18 (3.11, 3.25)
2	27.7 (26.6, 28.9)	3.65 (3.58, 3.71)	7.60 (7.42, 7.79)
3	25.3 (24.1, 26.5)	2.29 (2.25, 2.34)	11.0 (10.7, 11.3)
4 (High SES )	21.3 (20.2, 22.4)	1.81 (1.77, 1.85)	11.8 (11.4, 12.1)
**Unemployment**^**ll**^			
1 (Low SES)	27.7 (26.7, 28.7)	8.74 (8.62, 8.87)	3.17 (3.10, 3.24)
2	26.6 (25.5, 27.7)	3.56 (3.50, 3.62)	7.46 (7.29, 7.64)
3	24.3 (23.2, 25.5)	2.16 (2.11, 2.20)	11.3 (11.0, 11.6)
4 (High SES)	21.6 (20.4, 22.8)	1.21 (1.18, 1.25)	17.7 (17.2, 18.3)

^*^ Confidence interval.

^†^ Percentage of the total population 25 and over in the ZIP Code Tabulation Area (ZCTA) with any college attendance, 1 = smallest percentage with any college, 4 = largest percentage with any college.

^‡^ Percentage of housing units in the ZCTA with one or more persons per room, 1 = largest percentage with crowding, 4 = smallest percentage with crowding.

^§^ Per capita income in 1999 in the ZCTA, 1 = least income, 4 = most income.

^ll^ Percentage of the total population in the ZCTA civilian labor force that is unemployed, 1 = largest percentage of unemployed, 4 = smallest percentage of unemployed.

The magnitude of change in TB rates from low to high SES was much greater among the U.S.-born. Across the four SES-related measures, U.S.-born persons living in areas with the lowest SES had TB rates 4.8-7.2 times higher than those living in areas with the highest SES. Among the foreign-born, TB rates in the areas with the lowest SES were only 1.3-1.4 times higher than rates in areas with the highest SES. Rate ratios comparing TB rates among U.S.-born to those among foreign-born persons increased from the lowest to the highest SES level for all four measures of SES. For example, in areas with the highest proportion of crowded households (low SES), TB rates were 3.6 times higher among foreign-born compared to U.S.-born persons (95% confidence interval [CI]: 3.5, 3.7); in areas with the lowest proportion of household crowding (high SES), TB rates were 14.9 times higher (95% CI: 14.4, 15.4).

**Figure 1:**
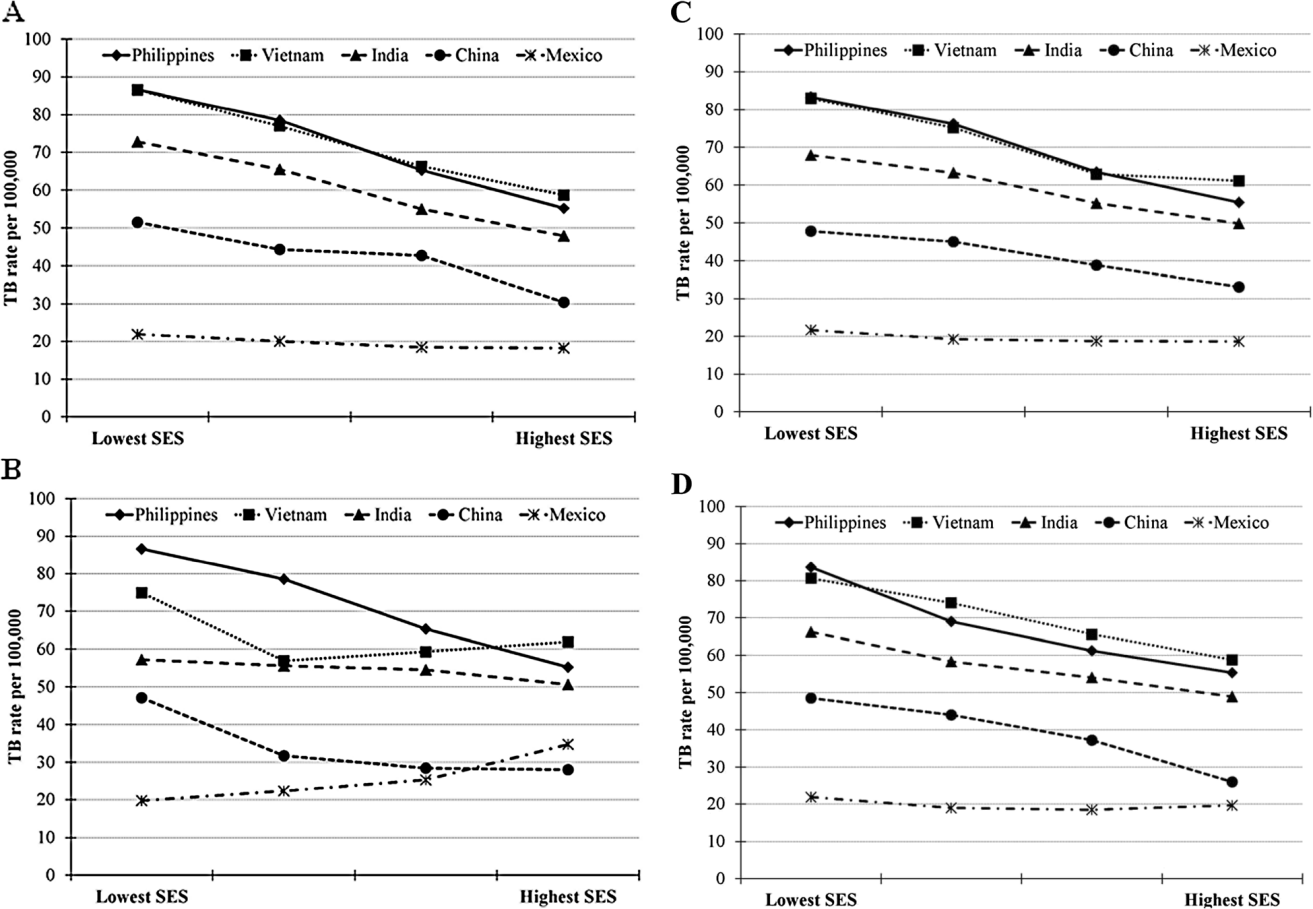
**TB rates by SES variables for foreign-born residents from the most common countries of origin.** Each panel illustrates the association between TB rates and a different U.S. Census 2000 ZIP Code measure of SES for persons born in the Philippines, Vietnam, India, China, and Mexico. The U.S. Census defines education (Panel **A**) as the percentage of the population 25 and older in the Zip Code Tabulation Area (ZCTA) with any college attendance; crowding (Panel **B**) as the percentage of housing units in the ZCTA with more than one person per room; income (Panel **C**) as per capita income in the ZCTA in 1999; and unemployment (Panel **D**) as the percentage of the total population in the ZCTA area’s civilian labor force that was unemployed in 1999.

TB rates per 100,000 population for the five most common foreign countries of origin were: Vietnam, 71.1 (95% CI: 69.4, 72.8); Philippines, 68.9 (95% CI: 67.5, 70.3); India, 55.0 (95% CI: 53.5, 56.5); China, 39.7 (95% CI: 38.4, 41.0); and Mexico, 20.5 (95% CI: 20.2, 20.8). For four of these five countries, evaluation of TB rates by the four U.S. Census SES variables showed the same association of higher TB rates with lower SES as in the overall analysis (Figure 1). A notable exception was the relationship between crowding and TB rates among persons born in Mexico, where TB rates were highest in the areas with the least crowding. At every SES level for each census variable, TB rates among persons from the Philippines, Vietnam, India, and China were higher than rates among foreign-born persons overall, while rates among persons from Mexico were generally lower (Table 2 and Figure 1).

### Multivariate analyses

In the Poisson model that included only U.S.-born persons, lower SES was associated with higher TB rates for all four census measures of SES (Table 3). Crowding and unemployment had the strongest association with TB rates. In the model of TB rates that included only foreign-born persons, higher percentages of crowding and lower per capita income were associated with higher TB rates. The SES-related measures had a weaker association with TB rates among the foreign-born compared to the U.S.-born. For example, the TB rate ratio comparing the areas with the highest and lowest percentages of crowding was 2.05 in the U.S.-born (95% CI: 1.92, 2.18) compared to 1.16 in the foreign-born (95% CI: 1.10, 1.21). The sub-analysis that compared only the highest and lowest quartiles, excluding the 15% of records in the middle quartiles for all four SES variables, resulted in only small changes in the rate ratios, and no change in their relative positions. The rate ratio for the crowding variable, for example, remained the largest, increasing from 2.05 to 2.18 among the U.S.-born and from 1.16 to 1.21 among the foreign-born.

**Table 3:** Multivariate rate ratios* comparing TB rates in areas of low (referent) and high SES among U.S.- born and foreign-born populations

	**Foreign-born(95% CI**^**†**^**)**	**U.S.-born(95% CI)**
Education^‡^	1.01 (0.94-1.08)	1.60 (1.48-1.73)
Crowding^§^	1.16 (1.10-1.21)	2.05 (1.92-2.18)
Income^ll^	1.14 (1.06-1.23)	1.16 (1.07-1.26)
Unemployment^**^	1.00 (0.94-1.07)	1.88 (1.75-2.02)

* Multivariate models were adjusted for all SES variables.

^†^ Confidence interval.

^‡^ Percentage of the total population 25 and over in the ZIP Code Tabulation. Area (ZCTA) with any college attendance.

^§^ Percentage of housing units in the ZCTA with one or more persons per room.

^ll^ Per capita income in 1999 in the ZCTA.

^**^ Percentage of the total population in the ZCTA civilian labor force that is unemployed.

## Discussion

Consistent with previous studies that linked TB and poverty [12-14], this study found higher rates of TB among U.S.-born and foreign-born persons living in areas identified by the U.S. Census 2000 as having low income, high crowding, less education, and high unemployment. However, this SES gradient was much less steep among the foreign-born, as evidenced by the large differences in TB rates across SES levels among the U.S.-born, and the comparatively smaller differences among the foreign-born. Compared to U.S.-born persons, rates of TB in foreign-born persons were high at all SES levels for all variables.

Two previous studies linked TB surveillance data with ZIP Code-level measures of SES from the U.S. Census 1990. One study, based on data from New Jersey, reported that foreign-born persons with TB resided in areas of higher SES than U.S.-born persons with TB [14]. The other study, which linked national TB surveillance data to ZIP Code SES measures, found that controlling for SES reduced racial disparities in TB rates more among U.S.-born than foreign-born persons [12].

The findings of this study support the hypothesis that TB rates among the foreign-born are more strongly influenced by experiences in their countries of origin than by their environments in the United States [5-7]. This study found that TB rates among foreign-born persons from the five most common countries of origin reflected the TB rates in their respective countries, both in relative magnitude and rank order: persons from the Philippines and Vietnam had the highest rates, followed by persons from India and China, with persons from Mexico having the lowest rates [7].

In addition, although the crowding variable had the strongest association with higher rates of TB in both foreign-born and U.S.-born populations, the association was weaker in the foreign-born. Household crowding can be considered both an indicator of low SES and a mechanism for TB transmission [12,20-22]. The weaker association between crowding and TB rates in foreign-born persons suggests a smaller role for recent transmission. Studies based on the genotyping of *Mycobacterium tuberculosis* strains have also concluded that TB among foreign-born persons is less frequently related to recent transmission than among the U.S.-born [6,23,24].

The association of SES with TB rates differed by country of origin. Rates among persons from Asia showed a relatively strong gradient with declining SES. In contrast, differences in TB rates by SES were much smaller among Mexicans, whose TB rates were highest in the least crowded quartiles and lowest in the most crowded. This discordance could be partly explained by differences in family structure, as other research has shown that foreign-born persons from Mexico often have larger households than other foreign-born populations, reflecting a higher proportion of married-couple families and a lower proportion of householders age 65 and older [25]. On the other hand, perception of crowding varies by culture; compared to Anglo-Americans and African-Americans, persons of Mexican and Vietnamese heritage have been shown to have a lower perception of crowding at every level of household density, regardless of income [26]. Future studies could explore the association of TB rates, SES, and country of birth in different regions of the United States, based on immigrant settlement patterns, which might shed light on these differences [27].

This study has a number of limitations, including the use of census ZCTA data based on ZIP Code, the only local geographic indicator available from the national TB surveillance database. ZIP Codes are used primarily by the U.S. Postal Service for delivering mail and are not necessarily reflective of neighborhoods; they are larger than census tracts and may encompass socioeconomically diverse areas [28]. Previous studies have found that SES measures at the levels of census tract and census block are more consistent measures of health disparities than SES measures at the ZIP Code-level [29]. In addition, U.S. Census 2000 ZCTAs are not always equivalent to ZIP Codes, so it is possible that some of the NTSS case ZIP Codes are incorrectly linked with ZCTA census data. Finally, while almost 96% of TB records of foreign-born cases had ZCTA matches, only about 75% of TB records of U.S.-born cases had matches; this could lower the rate estimates and affect the rate ratios in unknown ways. These discrepancies should be a strong stimulus for public health surveillance systems to switch from ZIP Codes to the collection of block group or census tract information.

An additional limitation is that measures of SES were calculated overall by ZCTA, not stratified on nativity, because this level of detail was not available. To the extent that this caused differential misclassification of SES, this could account for some or all of our findings.

A final limitation is the use of a static population denominator to estimate average TB rates over the ten-year period considered. These years were chosen to account for changes in the population before and after the 2000 U.S. Census.

The findings that (1) ZIP Code SES has a smaller impact on TB rates among foreign-born persons, and (2) this impact varies by country of origin, have implications for local TB prevention and control and for future research. Prevention and research efforts that focus only on identification and treatment of LTBI among low-SES persons or in low-SES areas will overlook or underserve some foreign-born populations. For example, TB control programs use enablers and incentives to promote treatment completion, which has historically been low [30]. To date, most studies of ways to improve treatment completion have been done in low-income groups and few have focused on foreign-born persons [30-36]; it is not known whether the impact of incentives and enablers varies by SES or country of origin. In addition, previous research has suggested that, depending on country of origin, some foreign-born populations are more likely than U.S.-born persons to receive TB care exclusively from private physicians [14]. Others have identified neighborhood disadvantage as an independent predictor of health care access, even after controlling for individual-level SES [37]. Such differences in access and care seeking highlight the need for collaboration between health departments and private providers, particularly with regard to the diagnosis and treatment of LTBI among the foreign-born.

## Conclusions

In the United States, to achieve the goal of TB elimination, TB programs and future research must include a focus on prevention through diagnosis and treatment of LTBI [9,10]. This will require increased understanding of the economic situations of foreign-born persons in the United States and the interactions between SES and country of origin. The traditional associations of TB with poverty and crowding are no longer sufficient to fully explain the epidemiology of TB in the United States or to meet the needs of all persons at risk of TB. TB prevention programs may need to attend to communities with high-risk foreign-born populations even in areas without relative SES deprivation. Therefore, TB programs and researchers should broaden their focus to include more socioeconomically diverse foreign-born populations.

## Abbreviations

TB: Tuberculosis; SES: Socioeconomic status; CI: Confidence interval; ZCTA: Zip Code Tabulation Area.

## Competing interests

The authors declare that they have no competing interest.

## Authors’ contributions

NAO collected the data, conducted the main analyses, and participated in the writing of the manuscript. ALD conceived the study, participated in the review and interpretation of the analyses, and edited the manuscript. CAW participated in the conduct, review, and interpretation of the analyses, and edited the manuscript. MPC participated in the conduct, review, and interpretation of the analyses, and edited the manuscript. JAG contributed to the concept development, reviewed draft versions of the results, made suggestions for the analysis, and edited the final version. DJK contributed to concept development, supervised the study, participated in review and interpretation of analyses, and led the writing. All authors read and approved the final manuscript.

## CDC disclaimer

The findings and conclusions in this report are those of the authors and do not necessarily represent the views of the Centers for Disease Control and Prevention.

## Pre-publication history

The pre-publication history for this paper can be accessed here:

http://www.biomedcentral.com/1471-2458/12/365/prepub
